# Acknowledgement to Reviewers of *Cells* in 2014

**DOI:** 10.3390/cells4010019

**Published:** 2015-01-07

**Authors:** Cells Editorial Office

**Affiliations:** MDPI AG, Klybeckstrasse 64, CH-4057 Basel, Switzerland

The editors of *Cells* would like to express their sincere gratitude to the following reviewers for assessing manuscripts in 2014:

Aguirre, Aitor
Ahern, Gerard P.
Akopian, Armen N.
Akutsu, Masato
Albert, Anthony P.
Almasan, Alex
Amendt, Brad A.
Andersson, R. P.
Anthony, Donald
Antonio Prieto, Jose
Aranda-Anzaldo, Armando
Banfield, Mark
Baragetti, andrea
Beech, David J.
Belibasakis, Georgios N.
Benichou, Giles
Bestard, Oriol
Bezprozvanny, Ilya
Bittel, Doug
Boulay, Guylain
Calarota, Sandra A.
Chan, Stephen Y.
Chang, Zhijie
Chen, Clark
Chen, Limin
Chen, Xuening Neal
Chen, Zhu
Cheng, Tong
Cho, Jeonghee
Chudley, Lindsey
Chung, Chin Ha
Claesson-Welsh, Lena
Clayton, Andrew
Cornell, Robert A.
Costa, Cristina
de Motes, Carlos Maluquer
Devaux, Yvan
DiRienzo, Joseph M.
Elemento, Olivier
Fabbri, Alessia
Fahrer, Jörg
Fairchild, Robert
Ferrari, Guido
Ferrari, Luca
Fierz, Beat
Fortin, Dale
Fox, Annette
Franco, Diego
Fujita, Wakako
Geddes, James W.
Ghislain, Michel
Ghosh, Asish K.
Gómez-Guerrero, Carmen
Göttlinger, Heinrich
Hafizi, Sassan
Harteneck, Christian
Hatziapostolou, Maria
Heldin, Carl-Henrik
Herington, Adrian C.
Hernández-Torres, Francisco
Hodny, Zdenek
Hoppe, Thorsten
Huan, Tianxiao
Ivey, Kathryn N.
Janetzki, Sylvia
Jang, Il-sung
Jeong, Keun-Yeong
Kaitsuka, Taku
Kawamukai, Makoto
Keane, Niamh M.
Keeble, Julie Elizabeth
Kelly, Melanie
Komada, Masayuki
Kuerten, Stefanie
Laforest, Brigitte
Lassot, Iréna
Lavoie, Brigitte D.
Lee, Won-Ha
Lindon, Catherine
Loll, Patrick
Loo, Deryk
Lorenz, Sonja
Lorenzo, O.
Matkovich, Scot J.
Matsumiya, Magali
Maurer, Ulrich
McArdle, Joseph
Mccarthy, John J.
Mericskay, Mathias
Metzinger, Laurent
Meyer, Florencia
Miletic, Hrvoje
Minden, Audrey
Minke, Baruch
Mirey, Gladys
Miyazawa, Masaaki
Mohamed, Salah A.
Monteith, Gregory R.
Montezano, Augusto
Mura, Ivan
Nakatsukasa, Kunio
Naya, Francisco J.
Ng, Shiyan
O'Donnell, Allyson F.
Oscarsson, Jan
Pabbidi, Reddy M.
Paviolo, Chiara
Pedersen, Stine Helene Falsig
Pedrazzini, Thierry
Perálvarez-Marín, Alex
Perraud, Anne-Laure
Pertovaara, Antti
Poon, Wayne W.
Preiss, Thomas
Rae, Mark G.
Ramachandran, Rithwik
Reinach, Peter S.
Ribeiro Macedo, Sandra
Rosato, Antonio
Rottembourg, Diane
Rubinstein, Jack
Sah, Rajan
Sakurai, Hiroaki
Salomon, David
Saotome, Masao
Sassano, Antonella
Schaefer, Michael
Schaper, Fred
Shaik, Shavali
Smits, Evelien L.j.m.
Snyder-Cappione, Jennifer E.
Sperling, Silke
Staehelin, Christian
Szallasi, Arpad
Teif, Vladimir B.
Thurston, Teresa L. M.
Tóth, Balázs István
Tsiokas, Leonidas
Veltel, Stefan
Vence, Luis M.
Verma, Rati
Vriens, Joris
Weitzman, Matthew
Whitcomb, Daniel J.
Wiesner, Silke
Woodman, Philip
Yamamoto, Janet
Yang, Hongmei
Yao, Yucheng
Yoshida, Yoshinori
Yudin, Yevgen
Zakharian, Eleonora
Zhang, Xu
Zheng, Fang
Zweit, Jamal

